# Epidemiology of Rubella Virus in a Fragile and Conflict-affected Setting – A Retrospective Analysis of 11 Years Case-based Data in South Sudan

**DOI:** 10.1093/ofid/ofaf221

**Published:** 2025-04-09

**Authors:** Sylvester Maleghemi, Atem Nathan Anyuon, Isaac Michael Zingbondo, George Awzenio Legge, Melisachew Adane Ferede, Patrick Freeman Eweh, Evans Mokaya, Patience Musanhu, Humphrey Karamagi, Sarah Wanyoike, Diana Chang Blanc, Olushayo Oluseun Olu, Ayesheshem Ademe Tegegne

**Affiliations:** Vaccine-Preventable Diseases Programme World Health Organization Country Office, Juba, South Sudan; Expanded Programme on Immunization Ministry of Health, Juba, Republic of South Sudan; Expanded Programme on Immunization Ministry of Health, Juba, Republic of South Sudan; Expanded Programme on Immunization Ministry of Health, Juba, Republic of South Sudan; Vaccine-Preventable Diseases Programme World Health Organization Country Office, Juba, South Sudan; Vaccine-Preventable Diseases Programme World Health Organization Country Office, Juba, South Sudan; Country Programmes Delivery Gavi Alliance, Geneva, Switzerland; Country Programmes Delivery Gavi Alliance, Geneva, Switzerland; Vaccine-Preventable Diseases Programme World Health Organization Country Office, Juba, South Sudan; Vaccine-Preventable Diseases Programme World Health Organization Intercountry Support Team, Harare, Zimbabwe; Department of Immunization, Vaccines and Biologicals (IVB) World Health Organization, Geneva, Switzerland; Office of the Director of Programme Management World Health Organization Regional Office for Africa, Brazzaville, Republic of Congo; Vaccine-Preventable Diseases Programme World Health Organization Country Office, Juba, South Sudan

**Keywords:** conflict-affected settings, rubella surveillance, rubella virus, South Sudan, vaccination

## Abstract

**Introduction:**

Since establishing routine immunization services in what was then known as Sudan in 1974, South Sudan has not yet introduced the rubella-containing vaccine into its national immunization schedule. This study aims to assess the burden of rubella infection within the existing measles case-based surveillance framework to provide evidence supporting advocacy for introducing the rubella-containing vaccine into the national immunization program.

**Methods:**

This study conducted a retrospective descriptive analysis of rubella infection using measles case-based surveillance data from 2013 to 2023. Data were analyzed with descriptive statistics and logistic regression using Epi Info, version 7.

**Results:**

During the study period, 17,987 suspected measles cases were reported, with 4944 serum samples collected. Of these, 2083 (42.1%) were positive for measles immunoglobulin M antibodies. Among 2861 samples that tested negative or indeterminate for measles, 678 (23.7%) tested positive for rubella immunoglobulin M antibodies. The study observed a significant increase in rubella positivity rates from 1.6% in 2014 to 34.4% in 2020. Logistic regression analysis showed that rubella infection was significantly more likely among children aged 5–9 years (odds ratio [OR] = 2.234; 95% confidence interval [CI]; 1.468–3.473, *P* < .001), 10–14 years (OR = 2.101; 95% CI, 1.570–4.428; *P* < .001), and 1–4 years (OR = 1.733; 95% CI, 1.149–2.687; *P* = .003), compared to children aged younger than 1 year (reference group). Rubella positivity was also slightly higher in urban settings than rural areas (OR = 1.139; 95% CI, 1.004–1.527; *P* = .034). Rubella cases demonstrated clear seasonality, with increased cases occurring from December and peaking in March.

**Conclusions:**

The study identified a high prevalence of rubella among young children, particularly those aged 1–9 years and in urban areas, highlighting the need for targeted vaccination strategies. These findings strongly support introducing the rubella vaccine into the national immunization program.

Rubella, also known as German measles, is a vaccine-preventable infectious disease caused by the rubella virus, an RNA virus from the Rubivirus genus within the Togaviridae family [1]. Although typically mild in children and young adults with symptoms including low-grade fever, rash, mild conjunctivitis, and occasional nausea, it poses a significant public health threat due to its severe teratogenic effects when contracted during pregnancy, particularly in the first trimester [2–6]. Congenital rubella syndrome (CRS), resulting from prenatal infection, includes severe outcomes such as deafness, blindness, heart defects, developmental delays, miscarriage, and stillbirth [7–9]. This stark contrast between rubella's mild presentation in individuals and its teratogenic impact highlights the critical importance of effective disease surveillance, strong vaccination programs, and targeted public health strategies to prevent prenatal infections [10, 11].

The availability of a highly effective rubella-containing vaccine, providing greater than 95% protection after a single dose, makes rubella a candidate for global eradication [1, 12]. Since the introduction of the rubella vaccines, many countries have observed substantial declines in rubella incidence. Despite its proven efficacy, vaccine uptake in routine immunization programs remains limited in Africa, with only 66% of African countries incorporating it into national schedules by 2020 [12].

Epidemiological data on rubella remain limited in several African countries, including South Sudan. Studies indicate high seroprevalence rates, reflecting widespread virus exposure despite limited vaccine adoption [13–18]. A systematic review covering 17 African nations reported natural immunity to rubella from 52.9% to 97.9%, highlighting considerable regional variation [18].

South Sudan, a conflict-affected country, established an integrated case-based surveillance system for measles and acute flaccid paralysis in 2011. Following World Health Organization (WHO) recommendations, this system also monitors rubella infections by testing measles-negative or indeterminate samples, a strategy similarly employed in countries like Ghana, Zambia, and Ethiopia [19–22]. However, there remains limited evidence on rubella epidemiology in South Sudan, hindering informed policymaking regarding vaccine introduction and rubella control strategies. Therefore, this study aims to analyze national measles surveillance data from 2013 to 2023 to characterize rubella epidemiology, identify vulnerable populations, and provide essential evidence to guide the potential inclusion of the rubella-containing vaccine into South Sudan's national immunization schedule.

## METHODS

### Study Design

**Figure 1.**
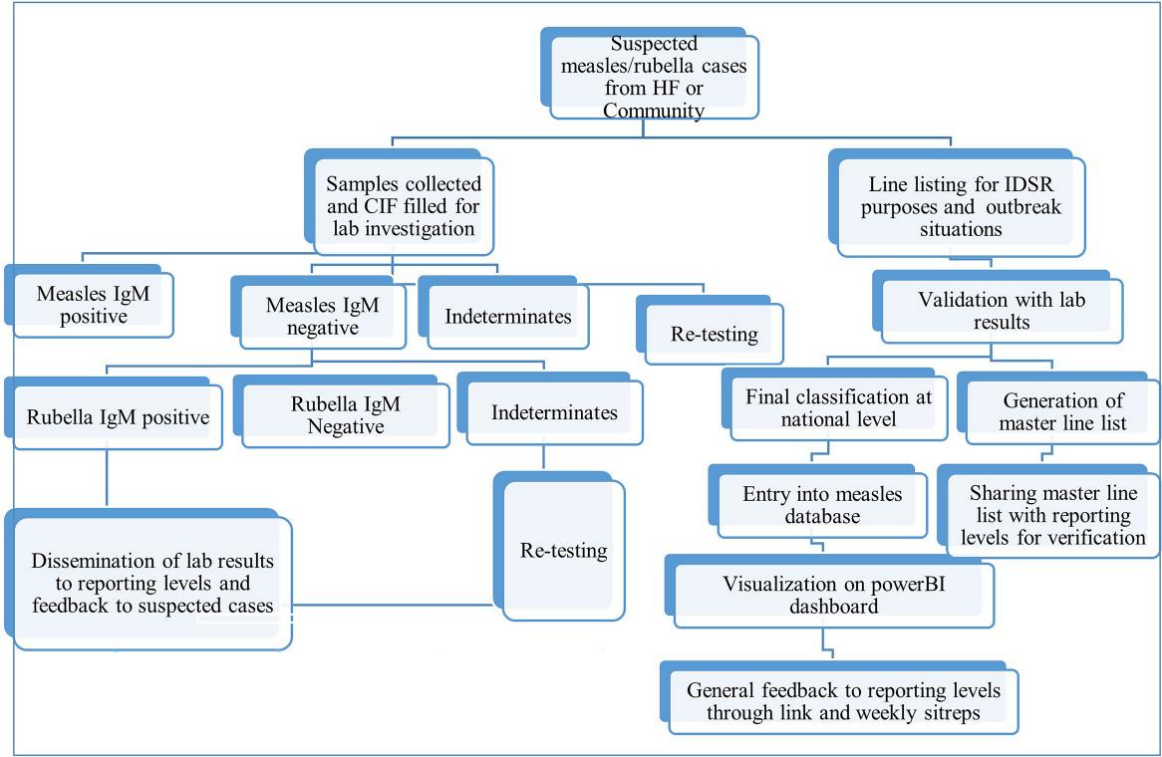
Measles and rubella surveillance and data management flowchart. CIF, case investigation form; HF, health facility; IDSR, Integrated Disease Surveillance and Response; IgM, immunoglobulin M.

Employing a retrospective design, this study analyzed existing surveillance data collected through South Sudan's national measles case-based surveillance program from 2013 to 2023. Serum samples initially testing negative or indeterminate for measles immunoglobulin M (IgM) antibodies had already undergone additional testing for rubella-specific IgM antibodies, following the established national protocol (Figure 1).

### Study Setting

South Sudan is a landlocked nation in East-Central Africa, bordering Ethiopia, Kenya, Uganda, the Central African Republic, the Democratic Republic of Congo, and Sudan. With a population of approximately 13 million, predominantly rural, the country is administratively divided into 10 states and 3 administrative areas [23]. Decades of armed conflict and instability have severely impaired socioeconomic development and weakened healthcare infrastructure, resulting in some of the poorest public health indicators in Africa. According to WHO/UNICEF estimates of national immunization coverage, the coverage of measles-containing vaccines is 72% [24], while life expectancy, maternal mortality, and under-five mortality rates are respectively 58 years, 1223 per 100,000 live births, and 99 per 1000 live births [25].

### The Measles-rubella Case-based Surveillance System in South Sudan

South Sudan implements a combined surveillance program for measles and rubella, which adheres to the unified strategy for controlling and eliminating these diseases. This program, endorsed by the Ministry of Health and supported by the WHO, follows the WHO Africa Regional Office (AFRO) measles/rubella surveillance protocol [26]. This protocol ensures consistent patient data and biological sample collection, contributing to a standardized approach to disease monitoring. The country's measles surveillance system demonstrated variable sensitivity and specificity in detecting measles cases, as reported in Ministry of Health weekly epidemiological reports, with improvements noted since 2020.

**Sample collection, serum separation, and storage:** In this measles-rubella case-based surveillance system, a 5-mL blood sample was collected by venipuncture from individuals of all ages and genders, meeting the WHO African Region's standard case definition for suspected measles [26]. After obtaining verbal informed consent, blood was drawn into anticoagulant-free tubes, allowed to coagulate, and centrifuged at 3000 rpm for 5 minutes to separate the serum. In facilities lacking centrifuges, blood samples were refrigerated until coagulation was complete. The serum was then aseptically transferred into sterile tubes and stored at 2 °C–8 °C until transported to the National Public Health Laboratory in Juba for testing.

**Laboratory analysis:** The laboratory stores the sera at −20 °C. The initial analysis involved testing for measles IgM antibodies within 7 days. Samples negative or indeterminate for measles are then tested for rubella-specific IgM antibodies using the enzyme-linked immunosorbent assay technique, following the manufacturer's guidelines (Enzygnost Anti-Rubella Virus/IgM kit; Siemens Erlangen, Germany).

**Case confirmation and data management:** Serum samples positive for rubella IgM antibodies are classified as confirmed rubella cases. Case-based forms documenting clinical and epidemiological data are completed, reviewed, cleaned, and entered into an electronic database managed by the Ministry of Health and WHO (Figure 1).

### Data Collection and Analyses for the Current Study

The data for this study were extracted from the national measles database, following WHO case definitions (Figure 1). Suspected measles cases were defined as individuals presenting with fever and generalized maculopapular (nonvesicular) rash or those suspected by a healthcare provider. Cases were excluded if consent was not obtained, incomplete or improperly labeled samples, or missing patient data.

The standardized case-based forms, which captured essential clinical and epidemiological information for all cases, were extracted and exported into an MS Access database. Descriptive epidemiological analyses were conducted using EPI Info (version 7, Centers for Disease Control and Prevention, Atlanta) to summarize the study population's prevalence and the epidemiological distribution of rubella and measles infections. Simple logistic regression models, which examined the association between rubella positivity and various demographic and clinical variables such as age, sex, geographic location, and symptom presentation, were constructed. The outcomes were measured using odds ratios (OR) with 95% confidence intervals (CI), providing insights into risk factors associated with rubella infection.

## RESULTS

### Descriptive Epidemiology

Between 2013 and 2023, South Sudan reported 17,987 suspected measles cases. Of these, 4944 serum samples were collected for diagnostic testing. Of the collected samples, 2083 (42.1%) tested positive for measles IgM antibodies, whereas 137 yielded indeterminate results. Further testing for rubella was conducted on 2861 samples that tested negative or indeterminate for measles IgM, identifying 678 cases (23.7%) positive for rubella IgM antibodies.

Notably, 2013 and 2020 recorded the lowest and highest rubella IgM positivity rates at 1.6% and 34.4%, respectively. The number of samples tested for rubella varied annually, with the lowest count (84 samples) in 2013 and the highest (488 samples) in 2019. This declined significantly from 488 in 2019 to 131 in 2020 and 176 in 2021 (Table 1).

**Table 1.** Distribution of Suspected and Confirmed Measles/Rubella Cases 2013–2023

Year	Total Suspected Measles/Rubella Cases Reported	Measles				Rubella				Total Suspected Measles/Rubella Cases Not Tested
		Total Samples Tested	No. of Positives	% of Positives for Measles IgM	No. of Indeterminates (%)	Total Samples Tested	No. of Positives	% of Positives for Rubella IgM	No. of Indeterminates (%)	
2013	642	257	173	67.3	14 (5.4)	84	7	8.3	5 (6.0)	385
2014	551	249	123	49.4	12 (4.8)	126	2	1.6	4 (3.2)	302
2015	390	232	55	23.7	3 (1.3)	177	47	26.6	6 (3.4)	158
2016	822	368	145	39.4	12 (3.3)	223	30	13.5	4 (1.8)	454
2017	395	305	90	29.5	18 (5.9)	215	71	33.0	23 (10.7)	90
2018	526	473	82	17.3	10 (2.1)	391	108	27.6	5 (1.3)	53
2019	2090	808	320	39.6	15 (1.9)	488	140	28.7	20 (4.1)	1282
2020	261	202	71	35.1	4 (2.0)	131	45	34.4	6 (4.6)	59
2021	205	193	17	8.8	8 (4.1)	176	28	15.9	2 (1.1)	12
2022	4149	855	393	46.0	12 (1.4)	462	157	34.0	3 (0.6)	3294
2023	7956	1002	614	61.3	29 (2.9)	388	43	11.1	34 (8.8)	6954
Grand Total	17,987	4944	2083	42.1	137 (2.8)	2861	678	23.7	112 (3.9)	13,043

Abbreviation: IgM, immunoglobulin M.

Fluctuations in the number of confirmed rubella cases were observed during the study period, with notable peaks in specific years. In 2015, there were 47 confirmed cases, followed by a significant peak in 2018 and 2019 with 108 and 140, respectively, and a decline in 2020 (45) and 2021 (28). In 2022, 157 confirmed rubella cases were recorded. Conversely, in 2023, confirmed cases decreased to 43, indicating a downward trend (Table 2).

**Table 2.** Rubella Confirmed Cases by Age and Year 2013–2023

Age Group	2013	2014	2015	2016	2017	2018	2019	2020	2021	2022	2023	Total
<1	2	0	2	4	3	9	7	3	0	5	0	35
1–4	0	2	17	14	33	62	71	15	11	53	14	292
5–9	4	0	22	6	24	31	35	16	11	56	21	226
10–14	1	0	6	2	8	4	23	9	5	29	6	93
15+	0	0	0	4	3	2	4	2	1	14	2	32
Grand total	7	2	47	30	71	108	140	45	28	157	43	678

**Figure 2.**
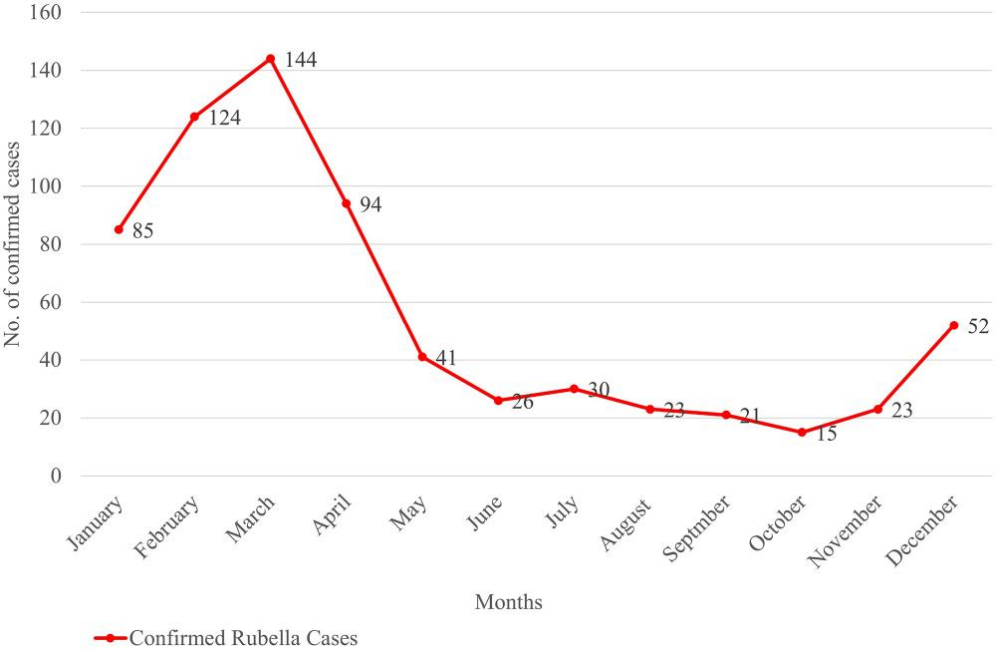
Monthly pattern of measles/rubella confirmed cases in South Sudan 2013–2023.

Monthly trends indicate a rise in rubella cases beginning in December, reaching a peak in March. February 2013–2023 reported 124 confirmed cases, marking it as 1 of the highest incidences recorded for any month throughout the decade. Mid-year months like June and July typically reported lower numbers, with June 2013–2023 documenting only 26 cases (Figure 2).

**Figure 3.**
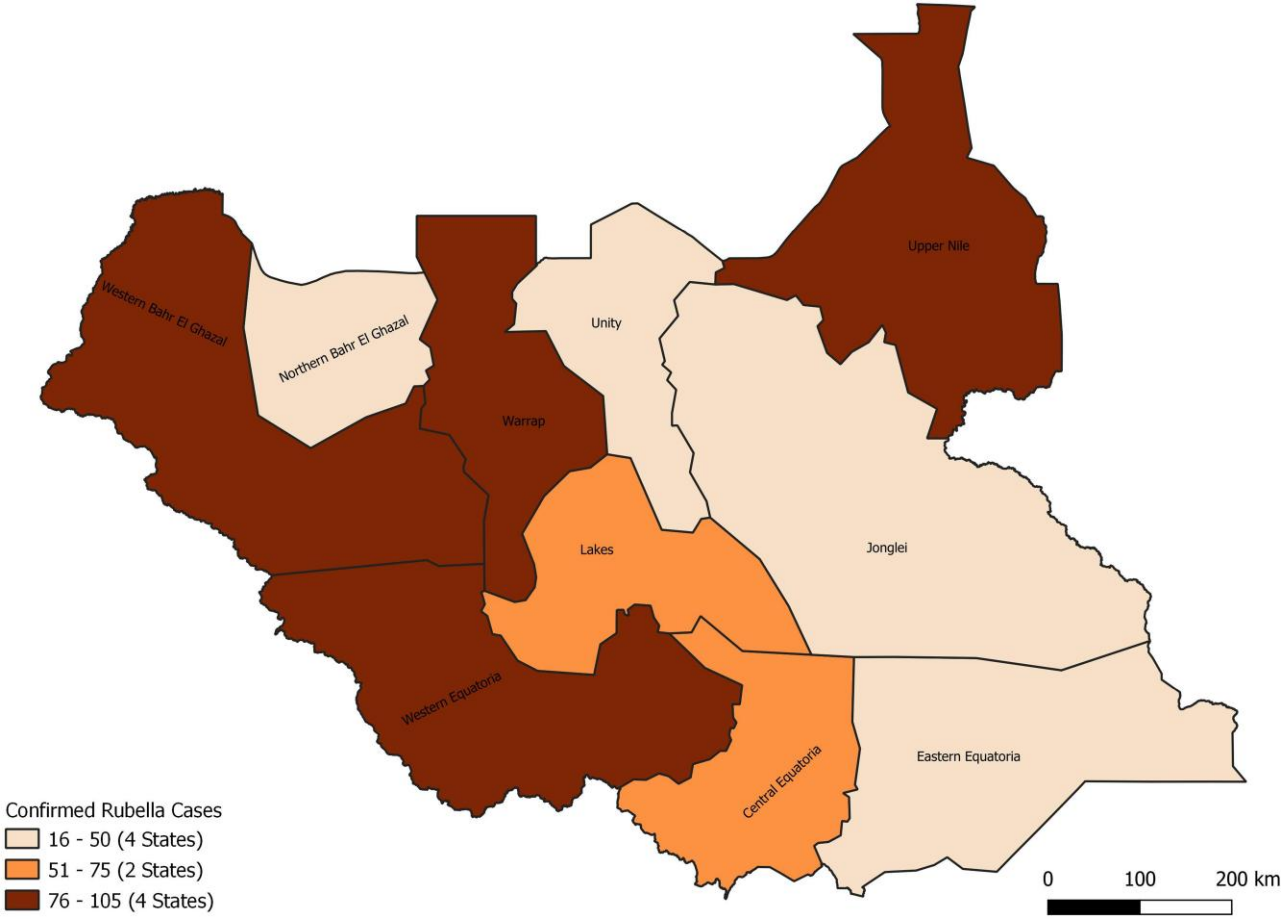
Distribution of confirmed rubella cases by states (2013–2023).

Geographically, the distribution of confirmed rubella cases exhibited concentration, with 59% arising from four states: Western Equatoria (15%), Western Bahr El Ghazal (15%), Warrap (15%), and Upper Nile (13%) (Figure 3).

However, the positivity rates were highest for Upper Nile (35.2%), Western Bahr El Ghazal (31.8%), Lakes (26.6%), and Warrap (25.8%) (Table 3).

**Table 3.** Distribution of Rubella Cases by State 2013–2023

State	Total Samples Tested for Rubella	No. of Positives	% of Positives for Rubella IgM
Central Equatoria	307	68	22.1
Eastern Equatoria	213	32	15.0
Jonglei	101	16	15.8
Lakes	263	70	26.6
Northern Bahr El Ghazal	304	48	15.8
Unity	215	43	20.0
Upper Nile	250	88	35.2
Warrap	403	104	25.8
Western Bahr El Ghazal	327	104	31.8
Western Equatoria	478	105	22.0
Grand total	2861	678	23.7

Abbreviation: IgM, immunoglobulin M.

Rubella cases were nearly evenly distributed by gender (51.3% male, 48.7% female) with no significant difference (*P* = .501) (Table 4). Additionally, the prevalence of rubella was slightly higher in urban populations than in rural areas, and the difference was statistically significant (*P* = .034) (Table 4).

**Table 4.** Statistical Distribution of Demographic Characteristics for Rubella

Age Group	Total Samples Tested for Rubella	No. of Positives	%	OR	95% CI	*P*
<1	245	35	14.2	Reference	…	
01–04	1115	292	26.2	1.733	1.149–2.687	.003
05–09	652	226	34.7	2.234	1.468–3.473	<.001
10–14	297	93	31.3	2.101	1.570–4.428	<.001
15+	552	32	5.8	0.772	0.541–1.746	.625
Sex	…	…	…	…	…	
Male	1469	348	23.7	0.621	0.451–1.235	.501
Female	1392	330	23.7	Reference	…	
Setting	…	…	…	…	…	
Urban	821	202	24.6	1.139	1.004–1.527	.034
Rural	2040	476	23.3	Reference	…	

Abbreviations: CI, confidence interval; OR, odds ratio.

The majority of rubella-positive cases were in children younger than age 15 years (95%), with the highest burden in the 1–4 and 5–9 age groups. The mean age of infection was 5–9 years. Infants younger than 1 year accounted for the lowest proportion (5%) (Table 5).

**Table 5.** Percentage of Positive Rubella Cases by Age 2013–2023

Age Group	Total Samples Tested for Rubella	No. of Positives	% of Total Cases Positive
<1	245	35	5
1–4	1115	292	43
5–9	652	226	33
10–14	297	93	14
15+	552	32	5

### Logistic Regression

The study identified the highest positivity rates within the 5–9 and 10–14 age groups, at 34.7 and 31.3, respectively (Table 4). Age-related logistic regression analysis revealed that rubella infection was more likely in children aged 5 to 9 years (OR = 2.234; 95% CI, 1.468–3.473; *P* < .000), followed by ages 10 to 14 years (OR = 2.101; 95% CI, 1.570–4.428; *P* < .001) then 1 to 4 years (OR = 1.733; 95% CI, 1.469–2.687; *P* < .003), and less likely in children 15 years and older (OR = 0.772; 95% CI, .541–1.746; *P* < .625) (Table 4).

## DISCUSSION

This study sought to characterize the epidemiology and transmission pattern of rubella in South Sudan from 2013 to 2023 by analyzing serological data from suspected measles cases subsequently tested for rubella IgM antibodies. The findings indicated a rubella positivity rate of 23.7% among samples retested after initial negative or indeterminate measles tests, suggesting a significant rubella burden within the country. This rate is higher than those reported in studies conducted in Ethiopia [27] but lower than the 37.6% positivity rate observed in Zimbabwe [28].

The increase in rubella cases detected during the study period likely reflects improvements in surveillance capacity and a true increase in rubella incidence. Surveillance enhancements included increased sample collection, integration of measles, and rubella detection into the established acute flaccid paralysis polio surveillance system, better-trained surveillance personnel, improved timeliness of reporting, and strengthened laboratory diagnostics. While these factors together improved detection rates, the upward trend in the percentage of confirmed rubella cases over the past 5 years, despite the absence of rubella-specific interventions in South Sudan, suggests a genuine increase in disease burden. However, without comparative data from neighboring countries with consistent testing practices, it is difficult to conclusively separate the impact of improved surveillance from a true rise in rubella incidence.

The temporary decline in rubella samples tested in 2020 and 2021 can be attributed to disruptions caused by the COVID-19 pandemic, including reduced surveillance activities due to lockdown measures and diminished healthcare-seeking behavior [29]. Furthermore, public health interventions intended to reduce COVID-19 transmission, such as the use of facemasks, lockdowns, social distancing, and limited travel, likely reduced exposure to the rubella virus, contributing to decreased positivity rates during this period. Similar trends were observed elsewhere in Africa and globally [30, 31].

Our study showed a seasonal trend in rubella incidence in South Sudan, with cases rising in December, peaking in March, and declining from May onward. This pattern is similar to findings from Zimbabwe, Cameroon, Ethiopia, and Côte d’Ivoire [31–35]. The peak months coincide with the hot season, suggesting that climatic factors may influence rubella transmission.

Understanding these trends is important for vaccination planning. Conducting mass immunization campaigns in the months preceding the seasonal surge (September–November) could help reduce transmission before peak incidence. Additionally, targeted public health messaging could improve vaccine uptake and encourage early healthcare-seeking behavior during this period.

Geographical disparities in rubella distribution are evident, with most cases concentrated in 4 states, primarily reported from their capitals. This concentration likely reflects population density factors that facilitate the transmission of the virus, particularly in urban areas where crowded living conditions and increased human interaction amplify the spread. Notably, the absence of Central Equatoria State, the country's capital, from the list underscores the need to strengthen surveillance efforts in its other counties outside of Juba County, which has already exceeded the set surveillance targets. Addressing these disparities requires targeted efforts to enhance case detection, particularly in underrepresented rural and peri-urban areas, to ensure a comprehensive understanding of rubella's distribution.

Our findings indicate that rubella cases were evenly distributed between males and females, consistent with findings from Ethiopia [22]. However, studies in Cameroon and Côte d'Ivoire reported differing gender patterns [33, 36]. These variations highlight the need for universal vaccination strategies rather than gender-targeted approaches. The difference in rubella prevalence between urban and rural populations was significant, with a higher prevalence observed in urban settings. Similar results were observed in studies conducted in Ghana and Tanzania [20, 37]. This finding challenges the common assumption that rural areas are more vulnerable, as noted in studies in Nigeria, China, and Turkey [38–40]. Additionally, a study in Cameroon from 2008 to 2014 found no significant association between rubella incidence and urban or rural settings [28].

The mean age of rubella cases in this study is similar to those with reports from other African countries before the introduction of the rubella vaccination [1, 41]. The highest burden was observed in school-aged children (5–14 years), consistent with studies from Ethiopia and Ghana [20, 27]. High positivity rates in the 5–14 age group, like findings by Lambert et al. and a recent Ethiopian study [1, 34], suggest that high social interaction in school settings facilitates transmission. In contrast, a study in Hangzhou, China, found the most affected group to be young adults (20–24 years) [42], highlighting regional differences in rubella epidemiology.

Early immunization provides individual protection and contributes to herd immunity. To maximize vaccine impact, routine immunization should target children younger than age 1 year, following measles-rubella vaccination strategies used elsewhere. Catch-up campaigns for older children (5–14 years) with the highest disease burden can help close immunity gaps. Given the higher prevalence in urban areas, initial campaigns should prioritize densely populated regions where transmission risk is greatest.

The findings of this study should be interpreted within the context of a few limitations. Gaps in surveillance data, underreporting, and possible misclassification of cases may have masked the true epidemiology of the rubella in the country. Additionally, rubella case detection primarily relied on the measles case definition, which may have resulted in missing several cases since many present asymptomatically. Not all suspected cases undergo rubella testing due to limited sample collection, prioritization of measles testing, and case classification protocols. Many measles-positive cases are not further tested for rubella, and in measles outbreak areas, only new epi-linked cases are listed, increasing the likelihood of undetected rubella cases. This limitation differs from broader case-finding challenges, as it specifically impacts the ability to distinguish rubella from measles within existing surveillance protocols.

### Future Research Directions

Given the limitations identified with the current approach of testing for rubella only after measles tests are negative, future research should focus on modifying the surveillance protocol to test all samples for both measles and rubella parallelly rather than sequentially. This testing strategy will provide more accurate and detailed insights into the transmission patterns of both viruses. Additionally, longitudinal studies are essential to assess the long-term effectiveness of rubella vaccination programs when introduced, particularly in reducing CRS cases. Such studies will help determine the impact of vaccinations over time and inform necessary adjustments to vaccination strategies to maximize public health outcomes.

## CONCLUSIONS

This study confirms rubella circulation across all states in South Sudan, with the highest burden in children younger than age 15 years. Cases in infants younger than age 1 year remain low, reinforcing the need to integrate a rubella-containing vaccine into the national immunization program.

The prospect of introducing a rubella vaccine holds promise for several reasons. First, it offers an opportunity to reduce the circulation of the virus within the population. By targeting children during their early years, when immunity is essential, the introduction of the vaccine can contribute to a reduction in rubella cases and its associated complications. Furthermore, this preventive measure carries a dual benefit: safeguarding the children and ensuring protection during their childbearing years.

Additionally, integrating birth defect surveillance, particularly for CRS, into the routine public health surveillance system is essential. Birth defect surveillance is currently unavailable in the country, leaving a gap in understanding the long-term impact of rubella virus circulation. Establishing such a system would provide evidence on the burden of CRS, supporting the introduction of the rubella vaccine.

## Notes

***Acknowledgments***. The authors sincerely thank the South Sudan Ministry of Health for their steadfast commitment to measles and rubella surveillance. We also acknowledge the essential support the WHO country office and WHO/AFRO provided in strengthening the national measles/rubella surveillance program in South Sudan. We thank the dedicated personnel at the National Measles/Rubella Laboratory for their invaluable contributions to laboratory testing. Last, we recognize and thank all healthcare professionals across South Sudan's healthcare institutions for their diligent work in case identification and sample collection, which significantly contributed to this research. The authors alone are responsible for the views expressed in this article, which do not necessarily represent the views, decisions, or policies of the institutions with which they are affiliated.

***Authors’ contributions.*** S.M. and A.A.T. conceptualized the study, collected and analyzed the data, and drafted the manuscript. A.N.A., I.M.Z., G.A.L., M.A.F., E.M., P.M., H.K., S.W., and D.C.B. participated in the manuscript writing and review, P.F.E. supported the analysis of results, and O.O.O. provided a critical review and made significant scientific contributions. A.A.T.’s and S.M.'s contributions are equal. All authors have reviewed the draft and approved the final manuscript for publication.

***Availability of data and materials*.** Most data elements for this study are presented in the tables and figures. However, the complete study data are available upon request from the corresponding author.

***Ethics approval and consent to participate.*** This study aimed to understand the epidemiology of Rubella in South Sudan and was integrated into the routine investigation of suspected measles cases. Blood samples were collected with informed consent from individuals, parents, or guardians in the case of minors. We adhered strictly to the ethical standards outlined in the Declaration of Helsinki regarding medical research ethics. Furthermore, all study protocols received approval from the Research Ethics Review Board (MOH-RERB) at the Ministry of Health, Republic of South Sudan. Additionally, administrative and ethical clearance to publish the data was sought from WHO/AFRO (WHO ePub-IP-00352247-PC). We affirm that all procedures were conducted according to these ethical guidelines.

***Financial support.*** No funding used for this paper.

***Consent for publication.*** Not applicable.
